# High-Fat Diet Alters Behavior and Hippocampal Gene Expression

**DOI:** 10.3390/ijms26189241

**Published:** 2025-09-22

**Authors:** Melissa S. Totten, Ava L. Peterson, Derek M. Pierce, Keith M. Erikson

**Affiliations:** 1 Chemistry, Biochemistry, and Nutrition Program, Salem College, Winston-Salem, NC 27101, USA; melissa.totten@salem.edu (M.S.T.); ava.peterson@salem.edu (A.L.P.); 2 Department of Nutrition, University of North Carolina Greensboro, Greensboro, NC 27412, USA; dmpierce5746@gmail.com

**Keywords:** hippocampus, high-fat diet, obesity, brain, amyloid precursor protein, alpha synuclein, brain-derived neurotrophic factor, anxiety, memory, gene expression

## Abstract

Consuming a high-fat diet (HFD) has been linked to gene expression alterations and negative behavior changes. The aim of this study was to evaluate the impact of a HFD on behavior and gene expression in the hippocampi of male and female mice from different strains to evaluate sex and genetic differences. C57BL/6J (B6J) and DBA/2J (D2J) mice were randomly assigned to either a control diet containing 10% kcal fat or a HFD containing 60% kcal fat for 16 weeks. Behavior was measured using the open field test for anxiety, nestlet shredding for general welfare, and novel object recognition for memory. Alpha synuclein (αSYN), amyloid precursor protein (APP), and brain-derived neurotrophic factor (BDNF) mRNA expression was assessed. The HFD led to reduced nestlet shredding for male B6J mice exclusively. There was a significant main effect of sex for fecal boli within the B6J strain and higher levels of fecal boli only for HFD male B6Js. No difference in memory was found in either strain. Significant three-way interactions between diet, sex, and strain for mRNA expression of aSYN and APP were found. However, the simple main effect of diet was only significant in the male B6J strain, revealing a 7-fold upregulation of hippocampal αSYN expression and 10-fold upregulation of APP in the HFD group compared to the control diet group. Although there was a significant strain by sex interaction effect for BDNF expression, there was no effect of diet on either strain. Overall, the HFD treatment impacted male B6J mice the greatest. This study demonstrates that biological sex and genetic factors should be considered when examining the impact of diet on behavior and the brain.

## 1. Introduction

Consuming a high-fat diet (HFD) and diet-induced obesity have been linked to negative behavioral outcomes such as compromised memory [1,2,3,4], increased anxiety [3,5,6], and decreased motivation [7]. Obesity and HFDs are also associated with brain disease [8] and gene expression alterations [9,10]. The impact of a HFD on the hippocampus is especially interesting, as this brain region is involved in key functions such as learning, cognition, and memory [11]. Genes that translate to proteins such as alpha synuclein (αSYN), amyloid precursor protein (APP), and brain-derived neurotrophic factor (BDNF) are expressed in the hippocampus and are correlated with many behaviors [12,13,14,15]. Overexpression of αSYN mRNA transcripts can lead to accumulation of αSYN protein [16] and interrupted function of the presynaptic SNARE complex, hindering the positioning and fusion of synaptic vesicles [17]. In the hippocampus, αSYN pathology has been linked to memory loss in patients with Dementia with Lewy Bodies [12]. Disturbances in normal gene expression of APP may lead to a build-up of beta amyloid, inciting conditions that promote neurodegeneration. The hippocampus is susceptible to beta amyloid protein aggregation due to high synaptic and metabolic activities in this brain region, which can trigger oxidative stress [18,19]. BDNF is a protein and growth factor associated with neuronal survival and brain plasticity [15,20]. The function of this neurotrophin in brain plasticity is related to learning, memory, and cognition in humans and rodents [15]. Currently, there are limited studies that address the impact of a HFD on αSYN, APP, and BDNF expression in the hippocampus and how gene dysregulation in this brain region may influence various behaviors in both males and females.

Obesity and HFDs have been associated with a higher prevalence of anxiety, as demonstrated in several human [6,21,22,23] and rodent studies [24,25,26]. However, there are other reports in humans and rodents that found no link between obesity and anxiety [27,28,29,30]. The relationship between obesity and anxiety is complex, often due to comorbidities and its bidirectional nature [6]. More research is needed to understand the impact of a HFD on anxiety and the influence of genetics and biological sex. Obesity can also have a negative effect on memory. In human studies, high body mass index (BMI) was associated with poor memory in adolescents [31], adults [32], and the elderly [33]. In rodents, several reports show a connection between a HFD or diet-induced obesity and memory decline [2,34]. A recent study using male B6J mice found that a HFD led to abnormal hippocampal morphology, impaired memory, and increased anxiety [3]. Although many studies show an association of obesity with reduced memory, few distinguish between males and females or differences in genetics that may cause discrepancies in the results.

Previously, our lab reported that a HFD was associated with a significant upregulation of hippocampal divalent metal transporter 1 mRNA and a significant downregulation of ceruloplasmin mRNA in male B6J mice but not in female B6J mice nor male or female D2J mice [35]. We also found that behaviors such as habituation [36], total distance travelled, and velocity [37] were reduced significantly in male B6J mice fed a HFD. The objectives of the current study were to evaluate the impact of a HFD on novel behavioral outcomes—including anxiety, general welfare, and memory, as well as on the gene expression of αSYN, APP, and BDNF in the hippocampi of male and female B6J and D2J mice from the same cohort. These inbred strains were selected based on their common use in comparative neuroscience research and their contrasting behavioral and physiological characteristics [38,39]. Furthermore, B6J and D2J mice are the parental strains used in the BXD recombinant inbred strain set for the GeneNetwork open-source project [40]. These strains have also been validated as suitable models for the study of diet-induced obesity [38,41,42]. We included both males and females to investigate the influence of biological sex on behavior and gene expression outcomes and to fill the literature gap of studies that mainly focus on only one sex. Based on results from our previous work, we hypothesized that behavior and gene expression would be impacted most significantly in male B6J mice fed a HFD.

## 2. Results

### 2.1. Male B6J Mice Fed a HFD Had Significantly Lower Levels of Nestlet Shredding

**Figure 1:**
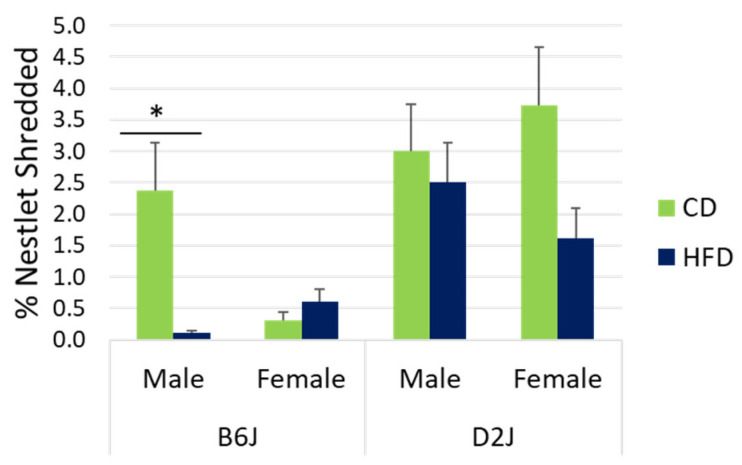
Nestlet shredding. Motivation, compulsivity, and welfare were assessed using nestlet shredding tendencies. There was a statistically significant diet effect for nestlet shredding in B6J males only. Data are represented as mean ± SEM. * *p* < 0.05. CD = control fat diet, HFD = high-fat diet, B6J = C57BL/6J strain, D2J = DBA/2J strain. The sample size was n = 9 for each treatment group, except for the D2J male HFD group, which was n = 8 due to failure to thrive for one mouse.

Nestlet shredding was measured as an assessment of motivation, compulsivity, and general welfare. Since the data within each treatment group was normal but did not pass the test for homogeneity of variance, an unequal variance *t*-test (Welch’s test) was used to compare differences in shredding between diet groups. Male B6J mice fed a HFD had 183% less nestlet shredding compared to male B6J mice fed a CD (t_8.041_ = 3.001, *p* = 0.017) (Figure 1). As an interesting observation, female D2J mice fed a HFD had 79% less shredding compared to the CD group. However, this observation did not reach statistical significance.

### 2.2. HFD Did Not Impact Center Entries in the Open Field but Did Impact Fecal Boli in Male B6J Mice

**Figure 2:**
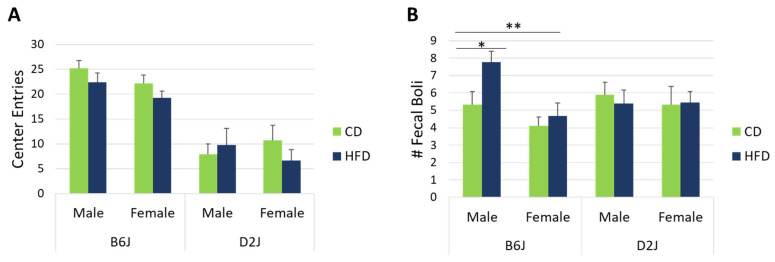
Center entries and fecal boli in the open field. Anxiety-like behaviors were assessed using center entries (**A**) and fecal boli (**B**) in the open field. (**A**) shows no statistically significant difference in center entries due to diet. (**B**) shows a sex effect in the B6J strain and a diet effect in male B6J mice for fecal boli. Data are represented as mean ± SEM. * *p* < 0.05; ** *p* < 0.01. CD = control fat diet, HFD = high-fat diet, B6J = C57BL/6J strain, D2J = DBA/2J strain. The sample size was n = 9 for each treatment group, except for the D2J male HFD group, which was n = 8 due to failure to thrive for one mouse.

Center entries are interpreted as being inversely proportional to anxiety-like behavior in rodents. In this study, we found no interaction effects nor main effects on center entries. Center entries for each treatment group are shown in Figure 2A. Fecal boli were counted at the end of each open field test as a second measure of anxiety-like behavior. Male B6J mice fed a HFD had 37% higher fecal boli compared to B6J mice fed a CD (F_1,63_ = 5.620, *p* = 0.021) (Figure 2B). There was also a significant main effect of sex within the B6J strain (F_1,63_ = 5.377, *p* = 0.024), with males showing 22% greater fecal boli counts compared to females. This effect of sex was greatest for B6J mice fed a HFD, with a 50% difference between sexes (F_1,63_ = 9.103, *p* = 0.004). There was no impact of diet or sex on fecal boli for the D2J strain.

### 2.3. HFD Did Not Impact Memory as Assessed by Novel Object Recognition

**Figure 3:**
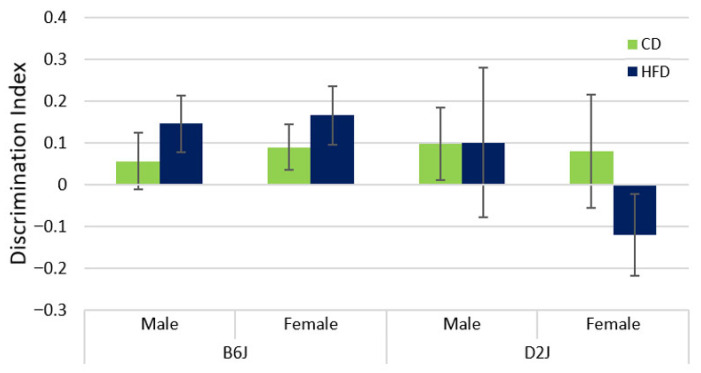
Novel object recognition (NOR). The NOR test was performed to assess the impact of diet, sex, and strain on memory. No statistically significant differences were found. Data are represented as mean ± SEM. CD = control fat diet, HFD = high-fat diet, B6J = C57BL/6J strain, D2J = DBA/2J strain. The sample size was n = 9 for each treatment group, except for the D2J male HFD group, which was n = 8 due to failure to thrive for one mouse.

The NOR test was performed to assess memory in male and female B6J and D2J mice. A two-factor ANOVA was used to evaluate the effects of diet and sex within each strain separately due to heterogeneity of variance between strains. We found no statistically significant main effects nor interactions for either strain. Interestingly, the discrimination index for female D2J mice was found to be negative, unlike the positive results for all other treatment groups (Figure 3). However, this result was not statistically significant.

### 2.4. HFD Significantly Upregulated αSYN and APP mRNA Expression in the Hippocampus of B6J Male Mice

**Figure 4:**
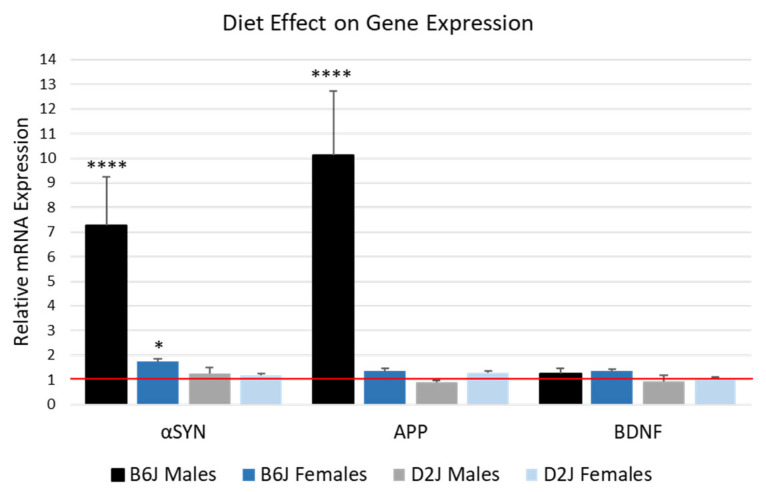
Impact of high-fat diet on gene expression in the hippocampus. The comparative Ct method was used to determine fold change in mRNA gene expression by comparing each CD group to the corresponding HFD treatment group. The red line represents the CD group for each sex and strain and the bars represent the HFD group for each sex and strain. Data are represented as mean ± SEM. * *p* < 0.05; **** *p* < 0.0001. Significance values are based on the simple main effect of diet for each strain and sex using the cycle threshold (Ct) values from the three-way ANOVA. *α*SYN = alpha synuclein, APP = amyloid precursor protein, BDNF = brain-derived neurotrophic factor, B6J = C57BL/6J, D2J = DBA/2J. Sample size was n = 3–5 for each treatment group. Outliers were removed based on boxplot analysis to ensure normality and homogeneity of variance.

There were significant three-way interaction effects between diet, sex, and strain for the mRNA expression of *α*SYN (F_1,28_ = 6.988, *p* = 0.013) and APP (F_1,30_ = 31.322, *p* < 0.0001). For *α*SYN, both male and female B6J mice fed a HFD showed a significant upregulation in mRNA, with a 7-fold increase in B6J males (F_1,28_ = 41.805, *p* < 0.0001) and a 1.7-fold increase for B6J females (F_1,28_ = 4.486, *p* = 0.043). The expression of *α*SYN in D2J mice was not impacted by the HFD. For APP, only the B6J male mice were significantly impacted by the HFD, with a 10-fold upregulation (F_1,30_ = 96.038, *p* < 0.0001). There were no statistically significant two-way or three-way interaction effects involving diet, nor simple main effects of diet on BDNF expression. The impact of the HFD on the expression of these genes is shown in Figure 4. Using the comparative Ct method, the CD for each sex and strain was set to 1, and HFD is expressed as fold change compared to each corresponding CD.

### 2.5. Female D2J Mice Express Significantly More BDNF in the Hippocampus Compared to D2J Males

**Figure 5:**
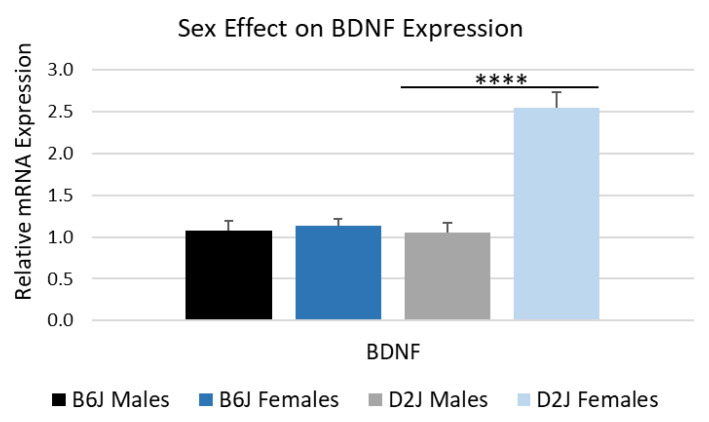
Biological sex effect on BDNF gene expression in the hippocampus. The comparative Ct method was used to determine fold change in mRNA gene expression by comparing males (control group) to females for each strain. Data are represented as mean ± SEM. **** *p* < 0.0001. Significance values are based on the simple main effect of sex within each strain using the cycle threshold (Ct) values from the three-way ANOVA. BDNF = brain-derived neurotrophic factor, B6J = C57BL/6J, D2J = DBA/2J.

Although there was no impact of diet on BDNF gene expression in either strain, there was a significant strain by sex interaction for BDNF expression (F_1,30_ = 14.642, *p* = 0.001). The simple main effect of sex within each strain was only significant in D2J mice (F_1,30_ = 32.377, *p* < 0.0001). The comparative Ct method was used to determine fold change in mRNA gene expression by comparing males (control group) to females for each strain (Figure 5). The D2J females expressed 2.54 ± 0.19-fold more BDNF compared to D2J males. There was no statistically significant sex effect for BDNF gene expression in the B6J strain.

## 3. Discussion

Diets high in fat have been linked to alterations in behavior [4,6,7] and gene expression [9,10]. In this study, using two murine strains, we found that a HFD significantly impacted nestlet shredding, fecal boli count, αSYN mRNA expression, and APP mRNA expression selectively in male B6J mice compared to female B6J mice and the D2J strain. These results align with our hypothesis that a HFD would impact male B6J mice the greatest based on our previous studies [35,36,37]. Although we expected a HFD to impair memory based on other diet studies using B6J mice [3,5], our results showed that diet did not influence memory in B6J and D2J strains based on the novel object recognition evaluation. For readers interested in the strain and sex comparison of weight gain over the 16-week diet treatment in this study, these data can be found in a previous report [43].

The nestlet shredding test has been used to evaluate a variety of behaviors, including obsessive–compulsive behavior [44], motivation or apathy [7], and general welfare [45]. It has also been used to measure the efficacy of anxiolytics [46]. Nestlet shredding for the purpose of nest building, warmth, or protection is a natural, spontaneous behavior in laboratory mice for both males and females [7,45,47]. Excessive shredding indicates repetitive, compulsive behaviors [44], while latency to shred can be a sign of apathy, depression, or poor health associated with disease progression [7,47]. In the present study, we reveal that a HFD was associated with a significant reduction in shredding behavior for male B6J mice (Figure 1). This latency to shred could indicate a lack of motivation caused by chronic exposure to a HFD or could be a sign of declining nervous system health. Although the nestlet shredding test has been suggested to have high sensitivity for assaying repetitive, compulsive-like behaviors in mice [44], our observation of reduced shredding in male B6Js may be a better indicator of generally compromised well-being or reduced mobility since nestlet shredding has low specificity. We also acknowledge that the results of our nestlet shredding test may have been impacted by the open field test and novel object recognition assessments that were conducted the previous week with this cohort of mice. Interestingly, impaired nestlet shredding has been linked to hippocampal dysfunction and neurodegenerative disease, specifically with mouse models of Alzheimer’s disease [47]. Furthermore, mice with hippocampal lesions have performed poorly in nest-building tasks and show a similar latency in shredding [48]. It is possible that the upregulation of αSYN and APP that we found in HFD-fed B6J male mice may be a sign of neurodegeneration in the hippocampus. Based on the data presented here and in our previous work with mice fed a HFD, we propose that a HFD acts as an environmental stress that can increase the risk for neurodegeneration and negative behavior transformations. This may explain the significantly decreased nestlet shredding that we found in B6J male mice.

There is evidence in humans [6,21,22,23] and in rodents [5,24,25,26] that suggests a relationship between obesity and anxiety. Recent studies using only male B6J mice found that a HFD led to increased anxiety-like behavior and hippocampal neuronal remodeling or neuroinflammation [3,49]. The hippocampus acts as a target of stress mediators and is closely related to anxiety modulation [50]. In the current study, we assessed anxiety in the open field arena by measuring avoidance of center entries and fecal boli quantity. Evading the center area and fecal boli are common behavioral assays for determining anxiety-like behavior in mice that are introduced to a novel environment [51]. Although we observed no statistically significant difference in center entries (Figure 2A), our data revealed a significant 37% increase in fecal boli produced by male B6J mice fed a HFD compared to mice fed a CD (Figure 2B). There was also a significant sex effect in the B6J strain, with males producing 50% more fecal boli compared to females (Figure 2B). In contrast to other murine strains, Keleher et. al. found that a HFD significantly increased fecal boli in both male and female SM/J mice fed a HFD compared to a CD group [52]. Grover et. al. found a significant decrease in open field fecal boli only for female TALLYHO/Jng mice fed a HFD compared to a chow diet [53]. These reported variations in fecal boli results between strains and sexes demonstrate the complexity of physiological changes that may occur due to consumption of a HFD based on genetics. Our fecal boli results may also be a reflection of gastrointestinal factors. However, during the weekly process of feeding and weighing the mice, we did not observe any major differences in fecal boli amounts between the different strains and sexes. Due to this uncertainty, we cannot draw any major conclusions about anxiety-like behavior from our fecal boli results. However, it is still noteworthy that the male B6J mice in our study were the only group that showed significant differences in fecal boli due to the HFD compared to B6J females and both males and females of the D2J strain.

Evaluation of center entries in the present study showed only a slight decrease for mice fed a HFD in most treatment groups, although this decrease was not statistically significant (Figure 2A). It is possible that the overall reduced mobility that we reported previously had an impact on central entries [37]. It is interesting to note the disparities in the scientific literature regarding center entries and percent center time for the assessment of HFD impact on anxiety-like behavior in B6J or B6 mice. For example, one study in male B6J mice found a negative correlation between weight gain and center entries [25], while others using B6 [28] or B6J [54] mice found no effect of diet on center entries. In a study with female B6 mice, there was no impact of diet on center entries after 12 and 21 weeks of HFD feeding, but mice fed a HFD had reduced center entries after 32 weeks of HFD feeding [26,55]. There are several factors that may explain these different results, such as the size of the open field, the age at which HFD is initiated, diet duration, and the use of different substrains of B6 mice. Although it is recommended by some protocols to test for anxiety within the first 5–10 min of the open field test [51,56], a wide variety of time frames are used in HFD studies. Considering the discrepancies in center time or center entry results in the diet studies listed here, it is possible that other behavioral assays for anxiety in rodents may be more accurate predictors of anxiety in obese mice or mice fed a HFD, such as the elevated plus maze, which has been used previously in HFD studies in mice and rats [57,58]. In future studies, we will consider using alternative evaluation methods for the study of anxiety-like behaviors.

In humans, a diet high in saturated fat can lead to memory impairment and an increased risk for Alzheimer’s disease [59,60]. A diet high in both fat and sugar may also impair hippocampus-dependent memory function [1]. In rodents, there are mixed results regarding the effect of diet-induced obesity or HFD on memory. For example, memory was compromised in young male B6J mice fed a HFD (60% kcal fat) for a duration of one week, with the diet treatment initiated at three weeks old [61]. A similar result was reported for middle-aged (11 months old) male B6J mice fed a HFD (60% kcal fat) for four months [62]. In contrast, male B6J mice fed a moderately HFD (32% kcal fat) at 11 weeks old for 6.5 months showed no difference in short- or long-term memory [63]. Female B6 mice at age 6–7 weeks old fed a 60% HFD for 12, 22, and 36 weeks also showed that diet had no impact on memory [26,55]. A recent study using both male and female B6J mice found that a HFD over five to six months led to impaired memory in males but not females, possibly due to the protective effects of estrogen [5]. Under normal diet conditions, there are natural strain differences in memory, which show that the B6J strain has superior memory compared to the D2J strain [64]. Our results show that a HFD did not have a statistically significant impact on memory as assessed by the novel object recognition test. This is consistent with one report of increased anxiety but no change in memory in male Wistar rats fed a HFD for 25 weeks [65] and another report that showed a 3-day HFD fed to female F344xBN F1 rats only impacted amygdala-dependent memory tasks but not hippocampus-dependent memory tasks [66]. Interestingly, a study with male B6J mice found that a 7-week HFD improved memory, but only in mice that did not display anxiety-like behavior [67]. There are alternative methods for evaluating memory and cognition that may provide a better understanding of the effects of a HFD on the hippocampus, including the Morris water maze test [34]. Moreover, the novel object test can be modified to change the spatial arrangement of objects rather than introducing a new object, which has also been shown to reflect neurobiological changes in the hippocampus. These alternative evaluations will be considered for future memory assessments in our laboratory. Future work could also include an evaluation of the prefrontal cortex for trace element dysregulation and gene expression alterations, as this brain region is also involved in memory, learning, and cognition.

For gene expression, we found that αSYN and APP mRNA were upregulated significantly in male B6J mice fed a HFD (Figure 4), but no significant change was observed in D2J mice. Our results showing dysregulated αSYN expression in the hippocampus due to HFD are consistent with other diet studies. For example, in a study using male ApoE^−/−^ and ApoE^−/−^/Tollip^−/−^ mice, HFD (42% kcal fat) led to the accumulation of αSYN and beta amyloid protein in the hippocampus and increased neuronal death [68]. Male B6 transgenic mice expressing human mutant [A30P] αSYN fed a HFD (45% kcal fat) demonstrated that HFD-induced obesity accelerates alpha-synucleiopathy and astrogliosis [69]. Furthermore, when m-Thy1-αSYN male mice fed a diet enriched with palmitic acid for three months were compared to a matched control (B6D2F1/J males), it was found that αSYN mRNA and protein were upregulated in the right hemisphere of the brain [70]. Most investigations related to the impact of a HFD on αSYN gene expression in the brain only used male mice or one strain. Our study provides novel information regarding the impact of a HFD on both males and females in two murine strains. The specific mechanisms of how dietary fat influences gene expression are still unknown but may be due to a combination of epigenetic modifications, disruptions in dopamine neurotransmitter systems, and changes in synaptic plasticity. In the hippocampus, αSYN can form protein aggregates called Lewy bodies, which are characteristic of Dementia and Alzheimer’s disease [12]. The results from our study suggest that the B6J strain, especially males, may be more susceptible to Lewy body formation in the hippocampus when fed a HFD. However, these results must be interpreted with caution since our study was limited by a small sample size and the recent discovery that gene expression throughout the left and right hemispheres of the brain may be asymmetrical [71].

APP is also highly expressed in the hippocampus of rodents [72]. Like the results we observed for αSYN, the effect of a HFD on APP expression had the most significant impact on B6J males, with a 10-fold increase in APP mRNA expression compared to the CD group (Figure 4). These results are consistent with our previous research, where we reported that a HFD significantly impacted gene expression of divalent metal transporter 1 and ceruloplasmin mRNA in the hippocampus of male B6J mice compared to female B6J mice and the D2J strain [35]. The current study is also consistent with other HFD studies involving male B6J mice that examined the impact of diet on the brain [73,74]. For example, one study using male B6J mice fed a diet based on palmitic acid from a period of weaning until 16 months of age found that beta amyloid protein accumulated in the hippocampus [73]. In another study using male B6J mice fed a diet based on milk fat for 22 weeks, mice fed a HFD had elevated APP expression in the hippocampus [74]. Unlike these previous studies, ours included both male and female mice, revealing a substantial difference between the sexes. We discovered that the hippocampi of male mice are impacted more than females in the B6J strain, and that the B6J strain overall is more impacted compared to the D2J strain. This sex-differentiated gene expression observation could be due to the fact that estrogen has the potential to suppress APP expression [75]. Increased APP promoter methylation in females may have the same protective effect [76]. In terms of strain differences, B6J and D2J mice have been shown to express genes differentially depending on the environmental exposure and tissue type [39,43,77]. Our study reveals the greater influence of a high saturated fat diet on the B6J hippocampus compared to the D2J hippocampus. Future investigations should include an evaluation of both protein and mRNA expression using the same brain hemisphere due to potential asymmetrical gene expression [71].

There are notable sex differences in the distribution of BDNF within different brain structures [78]. In rats, females usually have higher levels of BDNF in the hippocampus and cortex. In humans, there is no substantial difference in BDNF levels in the hippocampus, but females have higher levels of BDNF in the prefrontal cortex compared to males. Sex-differentiated BDNF expression in murine models shows mixed results [78,79,80]. In the current study, we found a statistically significant strain by sex interaction for BDNF expression. However, the HFD had no apparent impact on BDNF gene expression in either strain. Other diet studies in rodents show mixed results, with some reporting that a HFD was associated with reduced BDNF mRNA or protein expression [81,82,83] and others showing an increase in BDNF expression [84,85,86]. Most of these studies were performed with only one sex, usually with males. Some used whole brain tissue, and others evaluated the hippocampus specifically. Consistent with our results, Ferreira et. al. found no change in BDNF expression in the hippocampus of male B6 mice fed a HFD for 10 weeks. However, their study evaluated BDNF protein and not mRNA and only used one strain and one sex [87]. Although long-term consumption of a HFD tends to lead to decreased levels of BDNF, physical exercise has been shown to have a protective effect on BDNF expression [88,89]. It is possible that the HFD in our study had no impact on BDNF gene expression in either strain due to physical activity during the open field and nestlet shredding behavior tests in weeks 14 and 15, which occurred one week before brain tissue collection. It is also possible that these strains are resistant to the effects of a HFD in terms of BDNF expression. However, this would require a larger sample size to confirm this hypothesis. In terms of the significant strain by sex effect that we observed, D2J females expressed more BDNF compared to male D2J mice, regardless of diet type. There was no difference between BDNF mRNA expression in the B6J strain. Although many clinical and preclinical studies report higher baseline levels of BDNF in the female brain, sex-differentiated BDNF expression may depend on other factors, such as age, genetics, and experimental conditions [78,90]. For example, Matsuoka et. al. found that hippocampal BDNF mRNA in wild-type mice was higher in females compared to males at three months of age; however, no difference was found between the sexes at six months of age. They also found no difference in BDNF expression between males or females in 5xFAD mice at either age, demonstrating that mice of different strains have variable gene expression patterns [79]. When comparing wild-type to ZnT3 KO mice, McAllister et. al. found that BDNF mRNA expression was higher in the ZnT3 KO strain, but this difference was only observed in female mice [80]. In our study, we also observed greater mRNA BDNF expression in only one strain (D2J) and one sex (females). It is possible that there may have been differences in BDNF expression in the B6J strain at an earlier age that we missed. Our mice were approximately 19 weeks old at the time of tissue collection. Since neuronal estrogen is involved in regulating BDNF expression in females [90], future experiments should carefully monitor the estrous cycle and BDNF expression over time. We agree with a review from Chan et. al. that more studies are needed to investigate sex differences in BDNF expression across different strains, especially since the majority of studies published used males only [78].

In summary, the 16-week HFD treatment had the greatest overall impact on the B6J male mice in terms of nestlet shredding reduction, fecal boli production, aSYN upregulation, and APP upregulation. This is consistent with our hypothesis that male B6J mice would be impacted the most based on our previous reports [35,36,37]. The strengths of our study are the inclusion of both male and female mice to understand the role of biological sex and two different strains to compare genetics. The limitations of our study include the small sample size used for gene expression analysis, the lack of protein analysis with histological validation due to limited brain tissue, and the need for more robust behavior assessments, such as the Morris water maze or Y-maze to assess both short- and long-term memory and the elevated plus maze for anxiety. Our study can also be improved in the future by assessing behavior at multiple time points during the diet treatment. Although we followed a Latin square design for each behavior assessment and for tissue collection, we did not monitor the estrous cycle of the female mice, which could be a confounding factor for behavior and gene expression. Nevertheless, this study reveals interesting sex- and strain-dependent results regarding the influence of a HFD on the hippocampus and related behaviors in mice.

## 4. Materials and Methods

### 4.1. Animals and Diet

Male and female mice from strains C57BL/6J (B6J) and DBA/2J (D2J) (n = 36 per strain; n = 36 per sex) were received from Jackson Laboratory (Bar Harbor, ME, USA) at post-natal day 21. After a 3-day acclimation period in the animal care facility, mice were randomly assigned either a control diet (CD) containing 10% kcal fat (Research Diets, D12450J) or a high-fat diet (HFD) containing 60% kcal fat (Research Diets, D12492). Both diets were formulated with the same mineral mix (Research Diets, S10026B). Although both diets contained both soybean oil and lard as a source of fat, the HFD contained 52% kilocalories from lard, a significant source of saturated fat, compared to only 4% kilocalories from lard in the CD. Macronutrient and energy density comparisons for each diet formulation are shown in Table 1.

Ad libitum feeding was provided with 24 h free access to water. Mice were weighed once per week during the 16-week diet treatment period, and food weight was recorded three days per week. The mice were housed three per cage, with males and females positioned on opposite sides of the room. The temperature of the room was held at 25 °C and maintained on a 12 h light/dark cycle. Although the estrous cycle of female mice was not recorded, a Latin square design based on strain and sex was used for all behavior testing and tissue collection.

This study was conducted in an American Association for Laboratory Animal Care-accredited facility following a protocol approved by the Institution of Animal Care and Use Committee at the University of North Carolina Greensboro (protocol number 18-001). Procedures were performed by the principles and guidelines established by the National Institutes of Health for the ethical care and use of laboratory animals. One D2J male mouse assigned to the HFD group was humanely euthanized with isoflurane during week 10 of the diet treatment due to failure to thrive as determined by the animal facility veterinarian.

### 4.2. Behavior Testing

**Figure 6:**
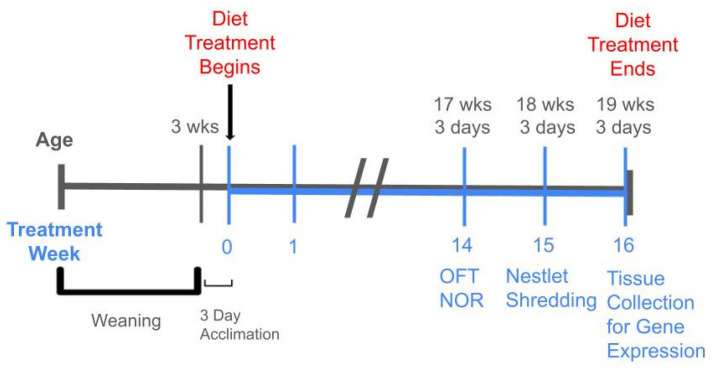
Experimental timeline. Male and female C57BL/6J and DBA/2J mice were received from the Jackson Laboratory after three weeks of weaning. After a 3-day acclimation period in the university animal facility, the control diet and high-fat diet treatment began. Mice were 3 weeks plus 3 days old when the diet treatment began. The open field test (OFT) and novel object recognition (NOR) test were conducted during week 14 of the diet treatment. The nestlet shredding test was conducted during week 15. Mice were euthanized for brain tissue collection after 16 weeks of diet treatment.

Behavior assessments included nestlet shredding for motivation, compulsivity, and welfare; the open field test with center entries and fecal boli count for anxiety; and novel object recognition counts for learning and memory. Details of each method are explained below. For all behavior assessments in this study, the sample size was n = 9 for each treatment group, except for the D2J male HFD group, which was n = 8 due to failure to thrive for one mouse, as explained above. Treatment weeks 14 and 15 were selected for the behavior assessments to provide sufficient time for any potential biological effects from the HFD to take place. Behavior test timing was also determined based on a previous investigation from our lab [10]. A timeline of the 16-week diet treatment with age and behavior testing is shown in Figure 6.

#### 4.2.1. Nestlet Shredding

Nestlet shredding tests were conducted during week 15 of the diet treatment for the evaluation of motivation [7], compulsivity [44], and overall welfare [45]. All mice were acclimated to the behavior test room for 30 min prior to assessment. Each nestlet was made from standard cotton material and measured 5.8 cm × 5.8 cm × 0.2 cm. Nestlets were acclimated to the behavior room for three days prior to testing to allow for adjustments to humidity. Each nestlet was then weighed on an analytical balance on the day that testing began. Polycarbonate mouse cages with a fitted filter-top cover were filled with fresh bedding to a depth of 0.5 cm. A single nestlet was placed in the center of each cage. Screening dividers were placed between test cages to avoid distractions from other mice. Each mouse was placed in a cage by itself with a nestlet and allowed to shred for 30 min. After each test, shredded nestlet material was carefully removed from each nestlet square. Nestlets were dried for 24 h and then reweighed to determine the degree of shredding. The results are reported here as percent nestlet shredded.

#### 4.2.2. Open Field Test

The open field test was conducted during week 14 of the diet treatment and was used to evaluate locomotion and anxiety. Our test design for the open field was based on published protocols [51,91] and the current literature [25,26,92]. Mice were acclimated to the behavior test room for a minimum of 30 min before each experiment. Clear acrylic 29 cm × 29 cm × 38 cm cubes covered with opaque white paper on all sides were used as the test arena. Cubes were cleaned with a disinfectant spray after each test and allowed to dry for 10 min before starting the next test. Recording software (TopScan Lite Version 2.00, Clever Systems, Inc., Reston, VA, USA) and video camera equipment were used for each recording. There were four separate test arenas (cubes) in the behavior test room, allowing for four mice to be evaluated at one time. Following the Latin square design, cube assignments were rotated to prevent proximity to the door from being a confounding factor. Mice had free access to food and water in their home cages but did not have access to food or water in the testing arena. All open field testing was conducted between 9 a.m. and 1 p.m. Mice were placed in the center of the cube at the start of each test, and activity was recorded for 30 min. At the end of each test, mice were transferred from the test cube to a separate polycarbonate cage to avoid inducing anxiety in the cage of remaining mice. Fecal boli were counted manually at the end of each test as one measure of anxiety.

Open field videos were analyzed using TopScan Lite Version 2.00, Clever Systems, Inc. Five-minute intervals were analyzed for each 30 min video to measure changes in behavior over time. Data from these videos were used to evaluate differences in center entries. A center zone of 30% was delineated to measure center entries (center entries are inversely proportional to anxiety-like behavior). The first five-minute time segment (0–5 min in the open field) was used for center entry analysis for the B6J strain, and the second five-minute time segment (5–10 min in the open field) was used for center entry analysis for the D2J strain. These specific time frames were selected based on the activity level of each strain.

#### 4.2.3. Novel Object Recognition

**Figure 7:**
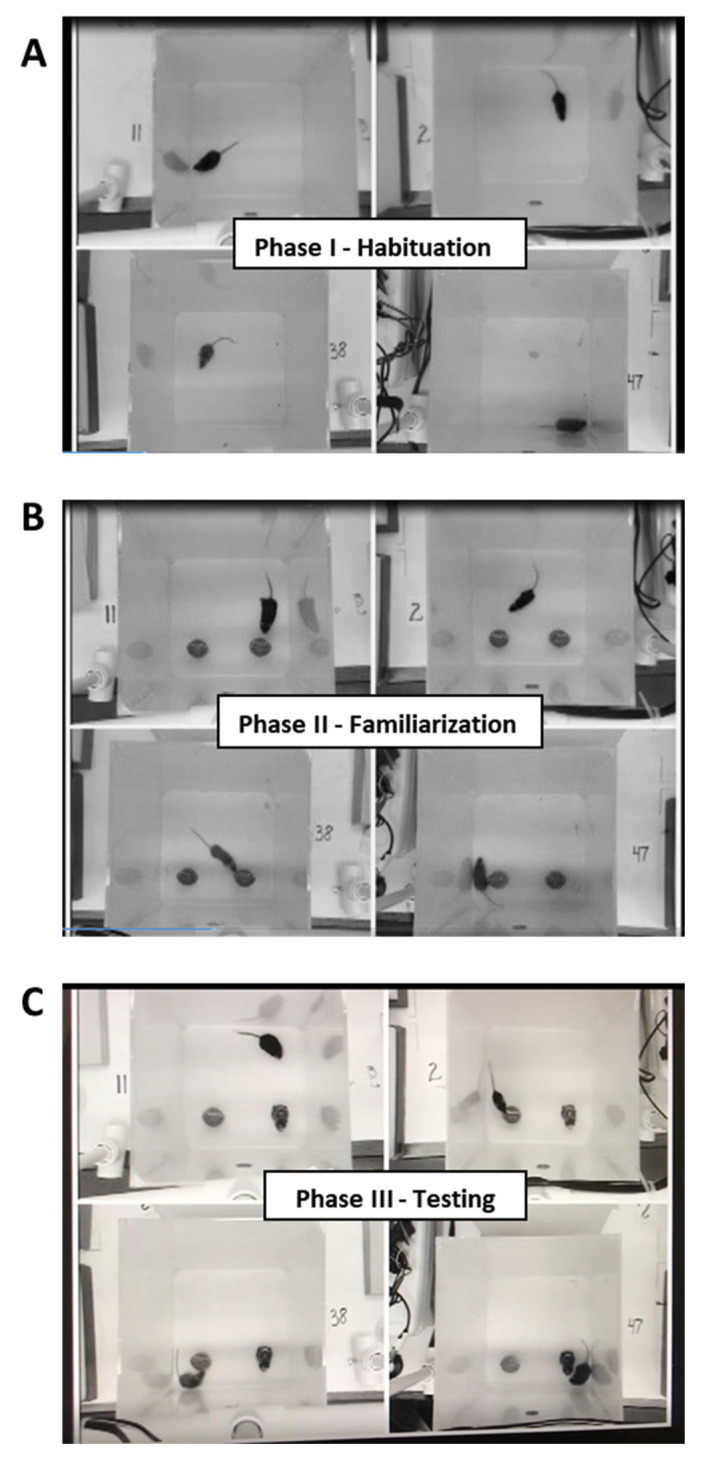
Novel object recognition test. Phase I (**A**) is the 30 min habituation phase for mice to gain exposure to the testing arena. Phase II (**B**) is the familiarization phase for mice to explore an identical object for five minutes. Phase III (**C**) is the testing phase for mice to explore either the familiar object or the new object for five minutes. Phase III occurs two hours after phase II and is used to evaluate memory.

The novel object recognition (NOR) test was used to evaluate memory and was designed based on published protocols [93,94]. This test consisted of three phases: habituation, familiarization, and testing (Figure 7). The open field test described above was used as the habituation phase (phase I). All NOR testing was conducted during treatment week 14 between 1 p.m. and 6 p.m., with each test starting approximately 24 h after the corresponding open field test. The objects used were nonporous figures of a similar size and color but a distinct shape. Objects were placed five centimeters from the back and side walls of each cube. Mice were placed in the cubes facing away from the objects on the opposite wall. During the familiarization phase (phase II), mice were introduced to two identical objects and allowed to explore for five minutes. Cubes and figures were cleaned with disinfectant after each test. The testing phase (phase III) began two hours after the familiarization phase. During this last phase, one of the familiar objects was replaced with a new object. Placement of the new object was alternated between the left and right sides of the cube to counterbalance any preference for cube location. Mice were given five minutes to explore the new and familiar objects during this testing phase.

Videos were analyzed manually for the entire five-minute video per mouse. Hand counters were used to determine the amount of time spent exploring each object. The mouse was considered exploring the object when the nose was touching the object. The results are reported here using the discrimination index as recommended in NOR protocols [55,93]. The formula used to calculate the discrimination index is as follows: (T_new_ − T_familiar_)/(T_new_ + T_familiar_). Rodents are naturally curious animals and will explore new objects as a normal process [95]. Therefore, a positive discrimination index value indicates normal behavior (exploring the new object more than the familiar object).

### 4.3. Tissue Collection

For this study, 44 mice were humanely anesthetized with isoflurane followed by rapid decapitation after the 16-week diet treatment (approximately 19 weeks and 3 days old). Tissue was collected using a Latin square design, following an order of one mouse from each group before repeating with another round. The specific order was as follows: B6J male CD, B6J male HFD, B6J female CD, B6J female HFD, D2J male CD, D2J male HFD, D2J female CD, and D2J female HFD. This cycle was repeated until all tissue collection was completed. The remaining mice were used for a separate study involving dopamine neurochemistry in the striatum [36]. Each brain was dissected sagittally into right and left hemispheres on an ice-cold stainless-steel platform. The hippocampus tissue was quickly isolated, frozen in liquid nitrogen, placed on dry ice, and stored at −80 °C. Left and right hemispheres were randomly assigned for gene expression analysis.

### 4.4. RNA Isolation and cDNA Synthesis

RNA was isolated from frozen tissue (n = 5 per group) using the RNeasy^®^ Plus Mini Kit (Qiagen Inc., Germantown, MD, USA) following the manufacturer’s protocol. Concentration and purity were confirmed using a NanoDrop™ 1000 spectrophotometer (Thermo Fisher Scientific, Inc., Waltham, MA, USA). Reverse transcription of RNA was attained on Applied Biosystems GeneAmp^®^ PCR System 9700 using Applied Biosystems High Capacity cDNA Reverse Transcription Kit (Life Technologies, Carlsbad, CA, USA) to prepare 20 μL samples for the thermocycler. Reaction conditions were as follows: 25 °C for 10 min, 37 °C for 120 min, 85 °C for five seconds, and 4 °C holding temperature at completion. Samples were stored at −20 °C until further evaluation.

### 4.5. Reverse Transcription Polymerase Chain Reaction (RT-PCR)

Relative gene expression was determined by RT-PCR on a 7500 Fast Real-Time PCR System from Applied Biosystems (Waltham, MA, USA) under the following conditions: incubation for two minutes at 50 °C, polymerase activation for two minutes at 95 °C, and 40 cycles of PCR (denature for three seconds at 95 °C and anneal/extend for 30 s at 95 °C). Taqman™ gene assays were supplied from Life Technologies (Carlsbad, CA, USA) and included the following: SNCA for alpha synuclein, APP for amyloid precursor protein, and BDNF for brain-derived neurotrophic factor. Each assay was prepared for RT-PCR using Applied Biosystems™ Taqman™ Fast Advanced Master Mix. The expression of each gene was normalized using 18S as the endogenous control. Normalized cycle threshold (Ct) values were used to determine variable interactions and main effects of diet, sex, and strain. The comparative Ct method was used to determine fold change in gene expression by comparing the CD group for each sex and strain to the HFD treatment group for each sex and strain. For some groups, outliers needed to be removed based on boxplot analysis to ensure normality and homogeneity of variance, resulting in a sample size of n = 3–5 per treatment group when diet, sex, and strain were compared (Table 2).

### 4.6. Statistical Analysis

The effects of diet, sex, and strain on behavior evaluations and gene expression were assessed using a three-way analysis of variance (ANOVA). Since the ambulatory characteristics of each strain differed significantly, a two-factor ANOVA was used for each strain with diet and sex as between-subject factors for the evaluation of center entries and NOR. Statistically significant interaction effects were evaluated further for simple main effects. In cases of no interactions, statistically significant main effects are reported. Differences between treatment groups at each level were determined by pairwise comparisons with a Bonferroni adjustment applied. Normality and homogeneity of variance of data were confirmed using the Shapiro–Wilk test and Levene’s test, respectively. Welch’s test for unequal variances was used for nestlet shredding. Boxplot analysis was used to determine outliers within treatment groups to ensure normality and homogeneity of variance. Statistical significance was accepted at *p* < 0.05, and differences were considered approaching significance between *p* = 0.05 and 0.10. Data are reported as means ± standard error of the mean (SEM). IBM SPSS Statistics 26 was used.

## 5. Conclusions

In conclusion, we found that a 16-week HFD treatment impacted male B6J mice more than female B6J mice and the D2J strain in terms of mRNA expression and general welfare. The most dramatic effect was the substantial upregulation of APP and αSYN mRNA in the hippocampi of male B6J mice fed a HFD. Although diet did not impact BDNF mRNA expression, we observed a significant strain by sex interaction, with D2J female mice expressing more BDNF compared to males, yet there was no apparent difference between sexes in the B6J strain. Additionally, the male B6J strain fed a HFD displayed compromised general welfare based on the nestlet shredding evaluation. We did not observe a significant influence of diet on memory. Although only male B6J mice fed a HFD had significantly higher fecal boli in the open field arena, we cannot conclude that this is related to anxiety-like behavior since these data were not supported by the open field center entry results. Future work should include estrogen monitoring, protein analysis, and oxidative stress measurement in the hippocampus to evaluate signs of neurodegeneration. More robust behavior assessments should also be incorporated for better translation to human behaviors. The intent of our study was to lay a foundation for future systems genetics-based studies. As a final takeaway, our study demonstrates important biological sex and genetic factors that should be considered when examining the impact of diet on behavior and the brain.

## Figures and Tables

**Table 1:** Macronutrient and energy density comparison of the control diet (CD) and high-fat diet (HFD) purchased from Research Diets, Inc.

Macronutrients	CD (10% kcal from Fat) D12450J	HFD (60% kcal from Fat) D12492
Fat	10% kcal	60% kcal
Carbohydrate	70% kcal	20% kcal
Protein	20% kcal	20% kcal
Energy Density	3.82 kcal/g	5.21 kcal/g

**Table 2:** Sample size per group for the diet–sex–strain analysis of alpha synuclein, amyloid precursor protein, and brain-derived neurotrophic factor mRNA gene expression.

Strain	C57BL/6J				DBA/2J			
Sex	Male		Female		Male		Female	
Diet	CD	HFD	CD	HFD	CD	HFD	CD	HFD
αSYN	4	3	4	5	5	5	5	5
APP	5	3	5	5	5	5	5	5
BDNF	5	5	5	5	5	3	5	5

Abbreviations: CD = control diet, HFD = high-fat diet, αSYN = alpha synuclein, APP = amyloid precursor protein, BDNF = brain-derived neurotrophic factor.

## Data Availability

Data described in this manuscript will be made available upon request. Please email the corresponding author, Melissa Totten: melissa.totten@salem.edu.

## Abbreviations

The following abbreviations are used in this manuscript:

APP	amyloid precursor protein
αSYN	alpha synuclein
BDNF	brain-derived neurotrophic factor
B6J	C57BL/6J
CD	control diet
D2J	DBA/2J
HFD	high-fat diet
